# Structural and functional brain damage in women with multiple sclerosis: A mini-review of neuroimaging sex-based studies

**DOI:** 10.3389/fneur.2022.1057446

**Published:** 2022-12-22

**Authors:** Antonia Ceccarelli

**Affiliations:** ^1^Department of Neurology, EpiCURA Centre Hospitalier, Ath, Belgium; ^2^Heart Rhythm Management Centre, Universitair Ziekenhuis Brussel - Vrije Universiteit Brussel, Brussels, Belgium

**Keywords:** multiple sclerosis, women, sex, MRI, brain, structural damage, functional damage

## Abstract

Neuroimaging literature in healthy humans has shown that there are sex-related differences in healthy brain's anatomical structure, associated function and susceptibility to neurological diseases. This mini-review summarizes findings derived from the current neuroimaging studies focused on sex-related brain structural and functional damage in women with multiple sclerosis (MS). MS is a chronic, multifactorial, immune-mediated disorder of the central nervous system that affects mostly women. Even if recent neuroimaging studies have shed light on distinctive features of sex-related MS differences in brain structural and functional damage, more research is needed to better elucidate sex-related MS pathological changes and susceptibility and to implement sex-tailored treatment strategies in MS.

## Introduction

Neuroimaging studies, in the last decades, have shown that there are sex-related differences in healthy brain's anatomical structure, associated function and susceptibility to neurological diseases (1–3). Conventional magnetic resonance imaging (cMRI) studies have suggested that men have larger total brain volumes accordingly to their larger body surface, larger white matter (WM) volume, larger volume of fronto-medial cortex, larger hippocampus and amygdala, while women seemed to have larger gray matter (GM) volumes (4). The latest developments of advanced MRI techniques have also shown sex-related differences in brain connectome and functional connectivity (4). These differences seem to be present already at birth and strengthen through the entire life span, under the influences of several factors, mainly hormones and genes. Moreover, increasing evidence is supporting sex-related susceptibility to diseases including neurological ones (3, 5).

Multiple sclerosis (MS) is a chronic, multifactorial, immune-mediated disorder of the central nervous system (CNS) characterized by different clinical courses (6). The most frequent clinical course is the relapsing remitting (RR), characterized by a neurological worsening followed eventually by a clinical recovery. Progressive courses such as secondary progressive (SP) and primary progressive (PP) ones are characterized by progressive worsening disability overtime (6). Interestingly MS affects mostly women at young age, but when touched, men present a worse disease, mainly a progressive course and more cognitive involvement (6, 7). Growing evidence is suggesting that sex has a role in multiple aspects of MS, including epidemiology, risk factors, clinical course, severity, comorbidities and in the structural and functional damage of the CNS (3, 7, 8).

Historically considered mostly as a CNS WM disease, lately, MRI imaging and pathological studies have disclosed that GM damage in MS is common, starts early in the disease course, progresses and worsens overtime and better correlates with the clinical and cognitive deficit (9).

This mini-review summarizes findings derived from the current neuroimaging studies focused on sex-related WM and GM brain structural and functional damage in MS. While recent neuroimaging studies have shown some sex-related distinctive features of brain structural and functional damage in MS, more research still is needed to better elucidate sex-related pathological changes and susceptibility and to implement sex-tailored treatment strategies in MS.

Post-mortem pathological, hormonal and genetical studies of sex-related changes in MS are not included in the present review, nor is a detailed analysis of each MRI modality and post-processing technique—these are beyond the scope of this review.

## Sex-related brain WM damage

This section highlights MRI studies on sex-related brain WM damage in MS.

At cMRI, WM damage includes WM lesions (WML) (T2 hyperintense, T1 hypointense, and contrast-enhancing lesions) that have been considered the hallmarks of MS. T2-hyperintense lesions (T2HL) are non-specific for the underlying MS pathology and show unreliable correlations with clinical status, but have ability to predict conversion from clinically isolated syndromes to clinically definite (CD) MS (10, 11). T1 hypointense lesions (T1HL), categorized as transient or chronic black holes correlate better than T2 hyperintensity with pathological changes reflecting mostly axonal loss and are more frequent in the progressive course of the disease (10, 11). Contrast-enhancing lesions (CEL) usually precede or accompany new T2-hyperintense lesion formation but disappear after an average of 3 weeks, and occur more frequently in the early stages of the MS disease. While they show poor correlations with disability, they are a useful marker for monitoring subclinical disease activity (10, 11).

Early cMRI studies have shown that women with MS (WMS) are more disposed to inflammatory lesions, while men with MS (MMS) present more chronic destructive lesions. In a pilot study of 50 naïve patients with CDMS, WMS were found to have significantly more CEL than MMS over a period of 3 years (12). Moreover, while no differences were found in T1HL, T2HL and T1/T2 ratio in a cross-sectional study of 138 CDMS patients, a trend toward a lower T1HL volume and a lower T1/T2 ratio was shown for PP WMS (13). In a cMRI study of 413 CDMS patients (14), WMS showed higher number of CEL and lower number of T1HL. In a study of 60 naïve RR patients (35 woman and 25 men) without hormone replacement therapy compared to matched controls, Tomassini et al. (15) confirmed that WMS have more CEL than MMS and lower testosterone concentrations than healthy women. However, in contrast with Pozzilli et al. (14), the study of Tomassini (15) failed to show that WMS had lower number of T1 lesions. In a double-blind, randomized, multicenter, placebo-controlled study (SPECTRIMS), in which 618 SPMS patients received IFN-b-1a 22 mg, 44 mg or placebo subcutaneously three times weekly for 3 years, a treatment-by-sex interaction effect was observed at follow-up on the burden of the disease and in the T2 activity measures (16). Indeed, WMS showed highly significant reductions, at both doses of IFNs, in the number of T2HL and percentage of active scans (16). In contrast with the above discussed studies, no sex-related differences were found in T2 and T1HL volumes, CEL and T1/T2 ratio in a large study of 763 undertreatment CDMS patients and 32 clinically isolated syndrome suggestive of MS (17).

Beyond cMRI, advanced MRI (aMRI) such magnetization transfer imaging, diffusion tensor imaging (DTI), magnetic resonance spectroscopy, phase imaging and relaxometry, have enriched our understanding of pathological correlates and the natural evolution of MS lesions. These techniques have also helped to characterize the diffuse abnormalities that escape detection by cMRI in the so-called normal appearing (NAWM) (10, 11), showing that NAWM damage is present in all MS phenotypes, starting from the earliest stage and spreading widely in the PMS. Nevertheless, despite the progress and availability of several aMRI techniques (10, 11) for characterizing WM structure and, despite the relevance of WM damage in MS, sex-related aMRI studies of WM damage are scarce. Overall, these MRI studies have confirmed the existence of sex-related differences in the WM, having less prominent changes in WMS than MMS, except for the WM atrophy that seemed more advanced in WMS.

Using DTI, Klistoner et al. (18), showed in a 1-year longitudinal study that progressive microstructural damage in lesions of the optic radiation was less prominent in WMS. Specifically, allowing the characterization of the preferential diffusion of water in WM, the integrity of tracts in specific neuronal circuits can be evaluated using DTI-based tractography and voxel-wise analysis (10, 11). Thus, in this study, Klistoner et al. (18) demonstrated in a group of 34 CDMS patients over a 1-year follow-up that WMS had developed less axonal loss and demyelination in the optic radiation's lesions than MMS, even when the comparison was adjusted for total brain lesion volume and optic radiation lesion volume, since MMS had higher lesion volume in the optic radiation than WMS at baseline (18). However, no sex-related differences in diffusivity changes were observed in NAWM of the optic radiation (18). In a subsequent DTI study (19), same researchers analyzed the evolution of chronic stable WML, peri-lesioned WM and NAWM in a group of 55 RR patients over 3.5 years. While worsening changes were observed overtime in the 3 target tissues, with a central to peripheral decreasing gradient, having worst changes in the core of the lesions, WMS showed less diffusivity changes in the lesions'core compared to MMS (19). Using DTI voxel-wise analysis, a study of 131 patients with CDMS 6 years post-diagnosis and with mild cognitive dysfunction, showed that the extent of diffusivity changes was sex-related. Changes were worse in MMS, especially in the measures of myelin integrity, and mostly located in posterior periventricular areas (20). Using a geostatistical approach, sex-related differences in WM lesion evolution were analyzed longitudinally over 3 years in 53 WMS and 36 MMS (21), showing that WML evolution can progress into 2 different patterns over time. The first pattern of WML evolution was longitudinally quasi-static, while the other one was worsening. Both sex group had WML with the 2 evolution patterns. Worse sex-related differences were greatly observed in the WML of the latter pattern in WMS (21).

High and ultra-high field MRI scans together with histopathological studies and aMRI techniques, such as phase imaging combined with dynamic contrast enhancement (22), that has recently emerged as a valuable method for investigating cells and microscopic tissues changes, have supported and expanded the notion that chronic lesions can evolve into an inactive scar or to a chronic active lesion in which the damage continues to progress over time (22). Chronic active lesions, also called smoldering lesions or slowing expanding lesions, can be identified pathologically and also using MRI, thanks to the presence of a curvilinear hypointensity along the edge of the lesion, the so-called paramagnetic rim that has specific pathological correlates (22, 23).

Chronic active lesions seems to be more frequent in the progressive course and in MMS, reenforcing the previously suggested concept that MMS suffer a more destructive disease than WMS (23). In a study of 39 patients with CDMS, using 7 Tesla susceptibility MRI techniques, WML with rims (14.1% of 846 total visible lesions at the susceptibility sequence) were more frequent in MMS (23). In the same study, MMS had a 10-times fold risk of having ≥1 rimmed lesions compared to WMS (23).

Few studies have looked at sex-related differences in WM volume suggesting that WM atrophy is more advanced in WMS (17, 24).

## Sex-related brain GM damage

This section highlights structural and functional MRI studies on sex-related GM damage differences. Thanks to aMRI techniques, such as double-inversion recovery for the detection of cortical lesions and the implementation of refined segmentation methods such as voxel based analysis for the detection of distinct topographical distribution of regional GM damage and atrophy, GM damage in MS has emerged lately as a new sensitive marker of disability, progression, and cognitive impairment, manifesting itself early in the disease course and affecting all MS phenotypes (25) and progressing over time (10, 11). Indeed, key indicators of GM diseases are cortical lesions, global and regional GM atrophy and diffuse GM tissue damage, involving cortical and subcortical structures beyond focal lesions (10, 11).

Sex-related MRI studies of GM damage in MS have showed that the GM is overall less damaged in WMS. In the study of Antulov et al. (17), normalized global GM volume and peripheral GM volumes were higher in WMS. Given the regional heterogeneity of the GM damage in MS, 89 MS patients (52 women and 37 men) were compared to 45 (28 women and 17 men) age matched controls in order to evaluate the effect of sex on regional GM atrophy, using voxel-based analysis approach. In this study, MMS and WMS were matched for age, disease duration, disability, T2HL count and volumes and proportion of treatment. There was also no difference in age between WMS vs. healthy women and MMS vs. healthy males (24). MS patients showed regional GM atrophy in the thalamus compared to matched controls. However, in addition, MMS showed greater regional GM atrophy in other structures such as putamen, precuneus, and medial frontal cortex as well as lower cortical thickness in the right intraparietal sulcus. Indeed, MMS had more widespread regional GM damage (24). Accordingly, WMS showed a more localized patter of regional GM when compared to MMS in the study of Sanchis-Segura (26). In this study, however, MMS exhibited additional decreased GM volume only in bilateral frontal areas. Sex-related MRI studies discrepancies regarding topographical distribution of regional GM atrophy may be related to sample size and MS clinical and demographics characteristics (24, 26). Using regional volumetry analysis, distinct topographical distribution of regional GM atrophy has been shown to differentiate MS patients also according to cognitive dysfunction (11). Building on this existing knowledge, Schoonheim et al. (27) analyzed the relationship between regional GM volumes of several subcortical GM structures and cognition in 120 CDMS patients (80 women) 6 years post-diagnosis and 50 age and educational level matched controls (30 women). No sex-related differences in EDSS, T1 or T2 lesion volumes, or disease duration were present, while the majority of the deep GM volumes were reduced in patients compared to controls, except for bilateral hippocampus, amygdala, and right nucleus accumbens in men, and right hippocampus and nucleus accumbens, bilateral amygdala, and putamen in WMS. GM volume reduction was lower on average in WMS. Furthermore, MMS also showed, as in previous studies (17), lower normalized global GM volume compared to WMS, but overall, deep GM atrophy showed the larger effect size of reduction. All cognitive domains, except the visuospatial memory, were affected in MMS, while none were significantly affected in WMS. Furthermore, sex, together with thalamic volume and education were overall the best predictors of cognition.

Sex-related differences also have been assessed over time in the progression of GM damage. In the study of Dolezal et al. (28), sex-related differences in MRI lesion and brain volumetric changes were investigated in a cohort of 181 RRMS patients, who were part of the Avonex-Steroid-Azathioprine clinical trial over 5-year follow-up period. No sex-related differences were observed in baseline and 5-year follow-up clinical characteristics and MRI lesion, global, tissue specific or regional brain volume. On note, in this study, at baseline subcortical deep GM, caudate, putamen, globus pallidus, thalamus and nucleus accumbens normalized volumes were significantly larger in MMS (28). In another study of a smaller sample size, after 6 years follow-up, a decreased regional subcortical frontal volume was observed in 25 RRMS WMS compared to 20 MMS matched for age, EDSS at onset, treatment, total brain volumes, GM, WM volumes and lesions volumes, while MMS showed at follow-up a decrease in total brain volume and total GM volume and greater percentage of global atrophy (29). Interestingly, in a total sample of 2,199 MS patients (female/male ratio of 1,651/548) age-matched followed through life, WMS, showed a greater proportion of RR course, lower lesion volume and greater GM and WM volumes from baseline to early and midlife. However, these differences were nullified in patients after 60-year-old, independently of the use or not of treatment (30). Likewise to Jakimovski et al. (30), another study strengthens the notion that sex-related differences can disappeared with older age (31). In this study, WMS in pre- and post-menopausal phase were compared to age matched men group. Interestingly, pre-menopausal WMS at the disease onset showed larger normalized total brain volume, larger normalized cortical volume and larger brainstem volume compared to MMS, while no sex-related differences were found in any of the GM volumes between MMS and post-menopausal WMS at the onset of the disease (31). Both studies (30, 31) suggested an important role of sex hormones on immune response.

To the best of our knowledge, only few studies have investigated sex-related changes of brain networks in MS (26, 32, 33). In more details, brain networks have been investigated with the use of functional MRI techniques to investigate brain plasticity as an adaptive or maladaptive mechanism in response to brain MS damage (34). Increased functional connectivity between different brain regions as a compensatory mechanism for their more extensive GM loss was found in MMS compared to WMS in the study of Sanchis-Segura et al. (26). In contrast, some other studies have found worse regional GM damage and related bigger network reorganization in WMS (32). By modeling single-subject intrinsic networks and quantifying subfield volumetric variations, sex-related differences in hippocampal vulnerability were found in a large 2 years longitudinal study of early RRMS (disease duration <5 years) patients compared to age matched controls (32). MMS and WMS were matched for disease duration, cognitive scores, T2HL volumes and hippocampal volume. Longitudinally MS patients remained cognitively preserved even if they accumulated hippocampal lesions. Functionally, at both baseline and follow-up, WMS displayed a more clustered hippocampal network organization and compromised regional integrity compared to MMS, that worsen over time for WMS. Furthermore, longitudinally, WMS developed an even more clustered network organization along with widespread regional tissue loss. Moreover, changes in hippocampal network and anatomical organization worsen overtime in WMS and were tightly related to cognitive performance, suggesting that sex influences cognition (32). Indeed, despite of the absence of sex-related differences in cognitive/memory performance over time, a strong interrelation between hippocampal network properties and cognitive/memory performance was found only in WMS (32).

Using resting-state functional MRI and graph analysis, functional connectivity between brain regions and network efficiency was explored in 60 MS patients compared to controls. Compared with age, disability, disease duration and lesion volume and matched WMS, MMS with reduced visuospatial memory showed decreased functional connectivity and network efficiency (33).

## Discussion and conclusion

This mini-review summarizes current literature of sex-related MRI differences in MS. The majority of the MRI studies seems to confirm the existence of sex-related differences in brain structure and function in MS, confirming, sex disparity observed in MS epidemiology, risk factors, genetics, course, clinical characteristics, hormonal and pathological changes (7, 8). Overall, despite of some discrepancies, WMS seemed to have less neurodegenerative brain changes than MMS that can partially explain clinical sex related differences, such as better prognosis, better relapse recovery, a lower risk for higher disability, for progressive course and for cognitive deficit. However, MS MRI sex-related changes still need to be fully investigated in order to better elucidate clinical implications and treatment response. Indeed, a recent study has shown that in healthy female brains, atrophy measures are influenced by hormonal changes suggesting that hormonal changes should be incorporate in MS MRI studies to discover better correlates of clinical changes (35). Interestingly, based on the current evidence, MS MRI sex-related differences seemed not to influence treatment response and vice versa (36, 37). However, till date, interventional trials have not been powered enough and have not been enough informative on clinical and MRI characteristics of sex groups to estimate sex-related clinical and MRI differences (37). Thus, sex-tailored MRI trials in MS are overall much needed.

In conclusions, sex-based MRI studies in MS are still scarce to draw final conclusions. Future studies using a large data set, a longitudinal design, a machine learning approach and correlating with hormonal changes, and treatment response, are warranted to better elucidate the role of sex in brain MS damage.

## Author contributions

AC conceptualized, designed, and wrote the mini-review.
